# Isolation and evaluation of *Cyberlindnera fabianii* strains to improve cigar tobacco leaves fermentation effect

**DOI:** 10.3389/fmicb.2024.1492042

**Published:** 2024-12-10

**Authors:** Sida Guo, Yasen Li, Zhen Yang, Qianying Zhang, Pinhe Li, Zhongrong Jiang, Jiabao Zhang, Yu Cao, Zhengcheng Zhang, Dongliang Li

**Affiliations:** ^1^Cigar Fermentation Technology Key Laboratory of China Tobacco, China Tobacco Sichuan Industrial Co., Ltd., Chengdu, China; ^2^Industry Efficient Utilization of Domestic Cigar Tobacco Key Laboratory of Sichuan Province, China Tobacco Sichuan Industrial Co., Ltd., Shifang, China

**Keywords:** cigar fermentation, *Cyberlindnera fabianii*, isolation, flavor components, sensory evaluation

## Abstract

**Introduction:**

Fermentation is a crucial process in cigar manufacturing. Exogenous addition of functional microorganisms for fortified fermentation can further improve cigar tobacco leaves fermentation quality.

**Methods:**

In this study, five *Cyberlindnera fabianii* strains were isolated from cigar tobacco leaves. Their fermentation effects were assessed by analyzing flavor components and major chemical compositions.

**Results:**

Among these five isolates, four *C. fabianii* strains demonstrated the ability to enhance the overall flavor profile, particularly by increasing esters and chlorophyll degradation products. Additionally, several strains, particularly F3 and F4, also reduced total alkaloid and nitrogen content. Among these, *C. fabianii* strain F4 exhibited the most significant improvements. Sensory evaluation revealed that strain F4 could enhance mellowness, richness and sweetness while reducing irritation and off-flavors in fermented cigar tobacco leaves.

**Discussion:**

Our findings indicate that *C. fabianii* strain F4 can significantly improve the overall quality of cigar tobacco leaves through fermentation. This study provides a promising microbial fermentation agent for potential application in the cigar industry.

## 1 Introduction

Tobacco is an important worldwide commercial crop for producing cigars, cigarettes, and other tobacco products (Cao et al., 2013). After undergoing a series of processes such as curing, fermenting, and rolling, cigar tobacco leaves (CTLs) are crafted into cigars (Jia et al., 2023). Fermentation is one of the crucial processes in cigar manufacturing. It can improve the internal chemical composition and smoking quality of CTLs (Ren et al., 2023). Through fermentation, macromolecular substances in CTLs (cellulose, proteins, etc.) that produce pungent and rough smoke during combustion can be degraded and converted into small molecular aroma components (Wu Q. et al., 2023). Traditionally, CTL fermentation is dependent on the microorganisms naturally present in tobacco leaves and their surrounding environment. In recent years, there have been many studies focused on characterizing the changes in microbial community composition during CTL spontaneous fermentation (Liu et al., 2021; Wu Q. et al., 2023; Zhang Q. et al., 2023, 2024). However, these fermentation processes are usually time-consuming. Additionally, the microbial communities on tobacco leaves are highly susceptible to external environment factors and difficult to control, leading to unpredictable quality variations across different batches (Zhang Q. et al., 2023). Exogenous addition of functional microorganisms for fortified fermentation has become one of the necessary methods to address these issues (Liu et al., 2015). Enzyme-producing and aroma-producing microorganisms have shown promising results in tobacco leaves fermentation, suggesting broad application prospects (Zheng et al., 2022b; Wu X. et al., 2023; Dong et al., 2024). While not strictly distinct, these microorganisms have different functional emphases. Enzyme-producing microorganisms secrete enzymes that effectively degrade macromolecules, reduce irritants. Aroma-producing microorganisms mainly synthesize flavor compounds directly through their metabolic processes, though they may also contribute to macromolecule degradation. Their synergistic action enhances the fermentation effect, improving tobacco quality (Wu X. et al., 2023).

Given their direct role in flavor synthesis, aroma-producing microorganisms have garnered particular attention in recent research (Yao et al., 2022; Zheng et al., 2022b). Various aroma-producing microorganisms have been utilized in CTL fermentation. For instance, *Candida* strains isolated from CTLs can reduce total alkaloid content while enhancing the concentration of flavor compounds (Jia et al., 2023). Acinetobacter can improve CTL quality by producing aldehydes and ketones (Zheng et al., 2022b). Additionally, recent investigations have demonstrated that aroma-producing microorganisms from diverse sources, such as Moutai aromatic microorganisms (Li et al., 2021) and various aroma-producing yeasts (Yao et al., 2022) also contributed to enhanced aroma compound content in CTL fermentation. However, the aroma-producing microorganisms that can be used in CTL fermentation still remain insufficient. More microorganisms need to be isolated for improving CTL quality.

*Cyberlindnera* is an aroma-producing genus, which can generate ester compounds (van Rijswijck et al., 2017). Due to this property, it has been applied in fields including microbial deodorization (Ma et al., 2023), baijiu and beer fermentation (van Rijswijck et al., 2017; Bellut et al., 2019; Liang et al., 2024). Previous studies have shown that ester can improve CTL quality by reducing irritation and providing a fruity, sweet aroma (Zheng et al., 2022a; Yang et al., 2024). Apart from generating ester compounds, *Cyberlindnera* can also produce a series of enzymes such as laccase (Olajuyigbe et al., 2019), tyrosinase, and manganese peroxidase (Danouche et al., 2021). These enzymes play a crucial role in the degradation of macromolecules in CTLs that cause irritation and off-flavor. To our knowledge, there have been no reports on isolating *Cyberlindnera* from tobacco leaves for CTL fermentation.

In this study, five *C. fabianii* strains were isolated from CTLs using the pure culture method. The isolated strains were individually inoculated into CTLs for fermentation. The strain with the best fermentation effect was selected based on major chemical compositions and flavor components. The fermentation effect of the selected strain was confirmed by sensory quality evaluation. The *C. fabianii* strain isolated in this study will be beneficial for accelerating the CTL substances transformation, and improving tobacco leaf quality.

## 2 Materials and methods

### 2.1 Materials and reagents

Ingredients used in this study were purchased from TIANGEN Technology Co., Ltd. (Beijing, China), unless noted otherwise. PBS solution (pH 7.2), Potato Dextrose Broth (PDB), agar powder, Ampicillin sodium, were purchased from Solarbio Technology Co., Ltd. (Beijing, China). Fungal genomic DNA extraction kit was purchased from Biospin Co., Ltd. (Hangzhou, China). QuEChERS extraction kit, QuEChERS SPE kit and other GC–MS required materials were purchased from Agilent (Santa Clara, United States). The cigar tobacco leaves for fermentation were collected and provided by China Tobacco Sichuan Industrial Co., Ltd. (Sichuan, China).

### 2.2 Isolation and identification of *Cyberlindnera fabianii* strains

To isolate *Cyberlindnera* strains from cigar tobacco leaves, 5 g CTLs sourced from Yunnan, Hubei, and Sichuan. were separately added into 200 mL PBS solution (pH 7.2) and under shaking conditions with rpm of 180, 30°C for 3 h. The mixture was filtered to remove CTL fragments, and the suspension was then spread on PDB plates (with ampicillin sodium). After incubation for 2.5 days at 30°C. The microorganisms with yeast colony morphology were isolated and cultured individually. The genomic DNA was extracted by fungal DNA kit and amplified with primer pairs ITS1 (5′-TCCGTAGGTGAACCTGCGG-3′)/ITS4 (5′-TCCTCCGCTTATTGATATGC-3′) and NL1 (5′-GCATATCAATAAGCGGAGGAAAAG-3′)/NL4 (5′-GGTCCGTGTTTCAAGACGG-3′) (Brysch-Herzberg et al., 2021). The PCR products of ITS and D1/D2 region was sequenced in Sangon Biotech (Shanghai, China), and the sequences were compared with the identified species using BLAST (Basic local alignment search tool). After identification, the isolated *Cyberlindnera fabianii* strain F4 was deposited in the China General Microbiological Culture Collection Center (CGMCC), Beijing, China, under accession number CGMCC 30447.

### 2.3 Cigar tobacco leaves fermentation

*C. fabianii* strains were cultured individually in PDB medium for 2 days at 28°C with 200 rpm shaking. The yeast cells were inoculated into 5 kg CTLs. The initial cell density of CTL fermentation was 1 × 10^6^ CFU/g *C. fabianii*, the initial water content was 30%. CTLs with same water content but without inoculation were used as control. Subsequently, all samples were fermented at 30°C (70% humidity) for 28 days and prepared for further testing.

### 2.4 Flavor component analysis of fermented cigar tobacco leaves

Flavor components of CTLs were analyzed with untargeted metabolomics techniques by gas chromatography–mass spectrometry (GC–MS), which has been reported previously (Hu et al., 2022; Jia et al., 2023). In brief, 2 g CTL powder were added into 10 mL water, after the powder was completely infiltrated by water through shaking. Subsequently, 10 mL acetonitrile and 50 μL phenylethyl acetate (10.477 mg/mL, internal standard) were added, the mixture was shaken for 2 h at 2,000 rpm. After frozen at −20°C for 10 min, QuEChERS extraction kit was used for metabolites extraction. A pre-formulated salt packet, containing 4 g MgSO_4_, 1 g NaCl, 1 g NaCitrate, and 0.5 g disodium citrate sesquihydrate was added and immediately shaken for dehydration. Subsequently, 1 mL supernatant was mixed with 0.15 g MgSO_4_ and shaken at 2000 rpm for 2 min. The supernatants were subsequently analyzed by GC–MS (DB-5MS column 60 m × 1.0 μm × 0.25 mm, 26–400 atomic mass units mass scan range). Helium served as the carrier gas with a flow rate of 1.2 mL/min. The GC oven started at 60°C, increased to 250°C at a rate of 2°C/min, then to 290°C at 5°C/min, and was held at the final temperature for 20 min. The MS operated in electron impact mode with a 230°C ion source temperature and a 70 eV ionization voltage. The detected compounds were identified by the NIST17 database.

### 2.5 Determination of major chemical components of fermented cigar tobacco leaves

The major chemical components of CTLs, including total alkaloids, total sugar, reducing sugar and total nitrogen were analyzed by continuous flow analytical system. The contents of total alkaloids were determined according to the Tobacco Industry Standard YC/T468-2013. The contents of total sugar and reducing sugar were determined according to the Tobacco Industry Standard YC/T159-2019. The contents of total nitrogen were determined according to the Tobacco Industry Standard YC/T161-2002.

### 2.6 Sensory quality evaluation

The fermented CTLs were rolled into 110 mm length, 14 mm diameter cigars. The cigars were balanced water content under a temperature of 20°C and relative humidity of 60%. Subsequently, the sensory quality was evaluated according to the Standard Evaluation Form provided by Great Wall Cigar Factory (Jia et al., 2023). Ten well-trained assessors who specialized in cigar production and evaluation were invited to conduct sensory quality evaluation. The sensory quality evaluation was conducted through two aspects: quality characteristics and flavor characteristics. For quality characteristics, 12 parameters (e.g., richness, matureness, irritation,) were rated on a 0–9 scale, where higher scores indicated better performance. As for the Flavor characteristics, such as bean, baking, nutty, etc., were evaluated using a 1–5 scale, with higher scores representing stronger flavor intensities. All panelists reached agreement on the evaluation scores for each sample.

## 3 Results

### 3.1 Isolation of *Cyberlindnera* strains

**Figure 1:**
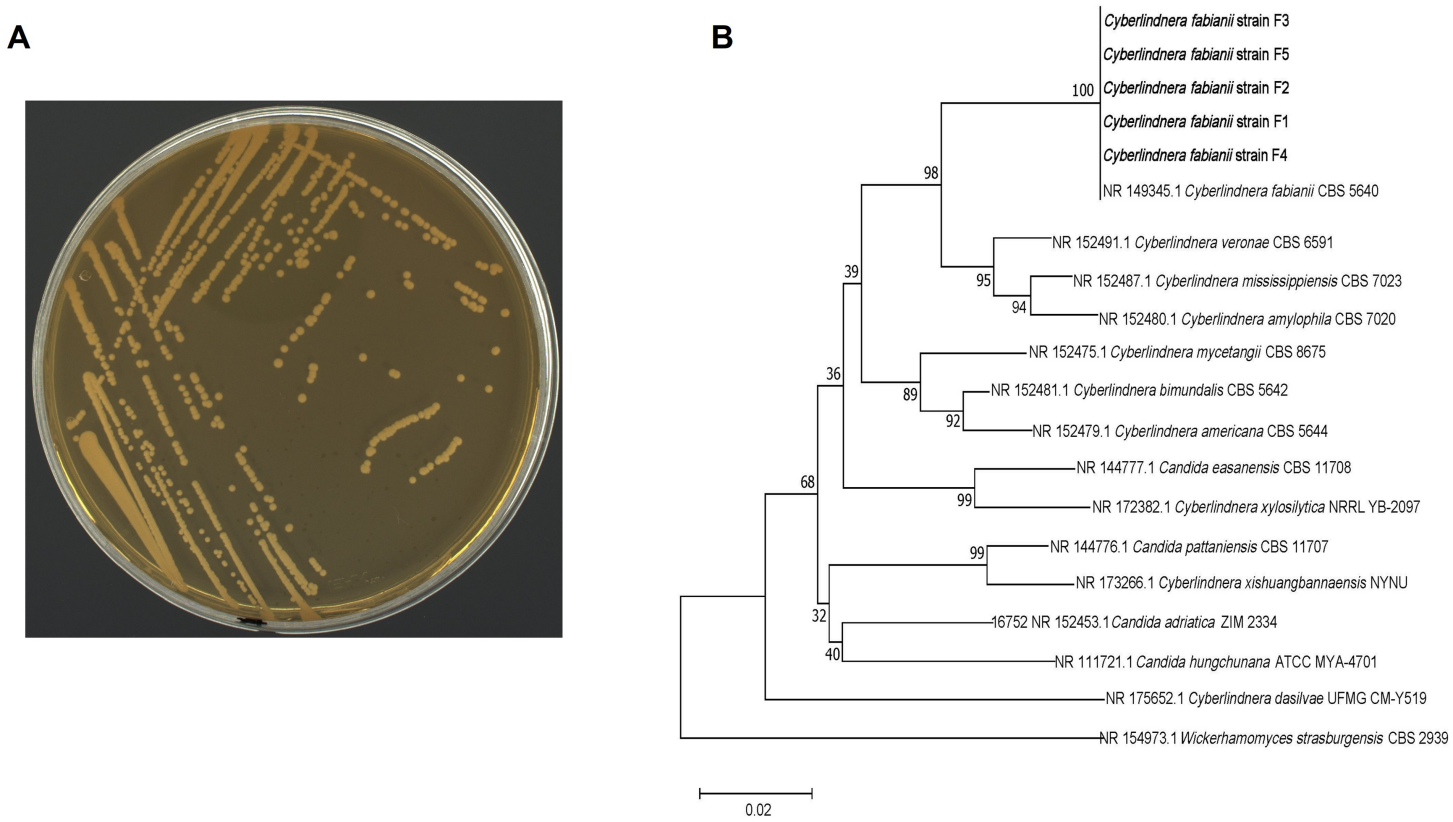
Colony morphology and phylogenetic analysis of isolated *C. fabianii* strains. **(A)** Colony morphology of isolated *C. fabianii* strain. **(B)** Phylogenetic analysis (tree) based on ITS sequence.

Previous study has demonstrated that *Cyberlindnera*, as an aroma-producing yeast strain, has the capability to produce ester compounds (van Rijswijck et al., 2017). This result indicates that *Cyberlindnera* had the potential to increase flavor components content through CTL fermentation. In this study, five *Cyberlindnera* strains with identical colony morphology (Figure 1A shows a representative strain) were isolated from domestic CTLs originating from Yunnan, Hubei, and Sichuan. The strains exhibited highest sequence similarities with *Cyberlindnera fabianii*, showing >99.26% identity in the ITS region and >99.64% identity in the D1/D2 region. These high sequence similarities strongly suggest that the isolated strains belong to *C. fabianii*. Phylogenetic tree of these isolated strains was constructed based on ITS sequence by MEGA11 (Figure 1B). The analysis revealed that five isolated *C. fabianii* strains (F1-F5) and *C. fabianii* CBS 5640 were in the same cluster, confirming their close genetic relationship. Interestingly, several *Candida* species were found to be interspersed within the *Cyberlindnera* clade, which suggests a close evolutionary relationship between certain members of the *Candida* genus and *Cyberlindnera*.

### 3.2 Comparative analysis of flavor components

**Figure 2:**
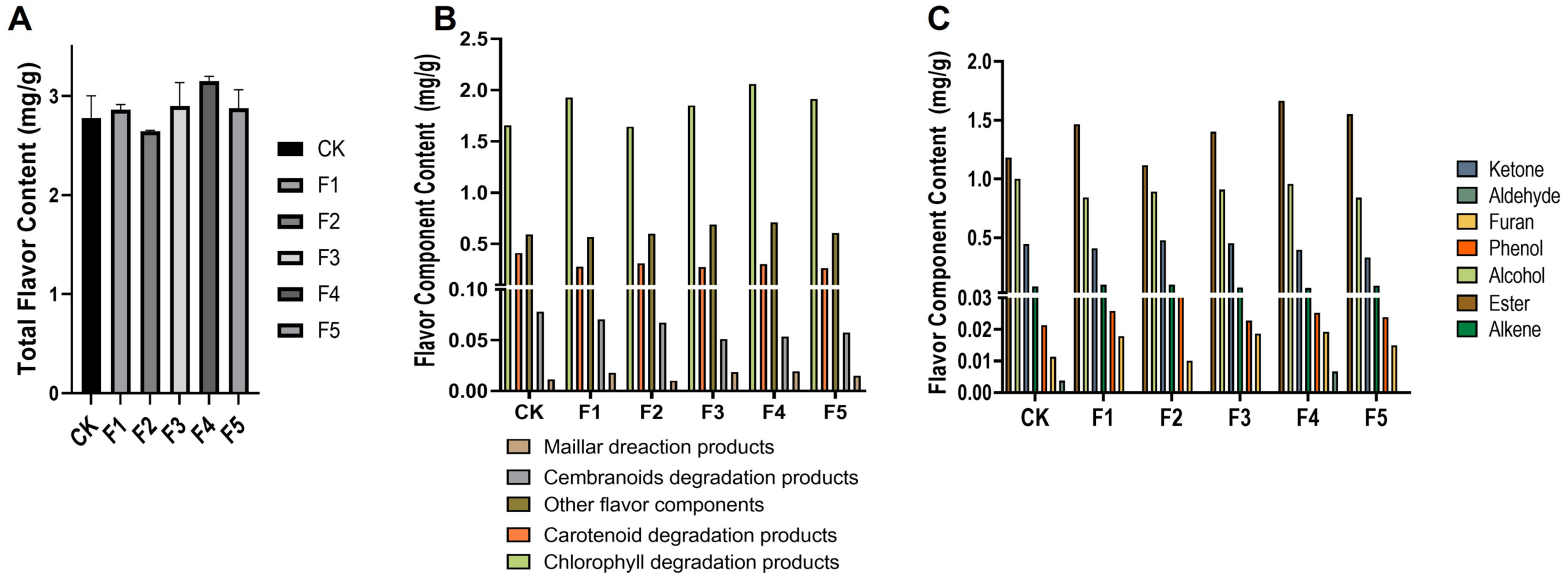
Flavor component content of CTLs after *C. fabianii* strains fermentation. Total flavor content **(A)**, distribution of flavor component content according to precursors **(B)** and chemical classes **(C)**. CK represents water fermentation control group, F1 ~ F5 represent different *C. fabianii* fermentation groups.

The flavor components determine the aroma profile of CTLs, which is an essential impactor of cigar quality (Hu et al., 2022; Zhu et al., 2024). To assess whether isolated *C. fabianii* strains can increase flavor component content in CTLs during fermentation, we analyzed the fermented CTLs using GC–MS. The metabolites were classified by precursors (Figure 2B) and chemical classes (Figure 2C). Based on the total flavor component content in CTLs after fermentation by *C. fabianii* strains (Figure 2A), the fermented groups were ranked from highest to lowest as follows: F4 > F3 > F1 > F5 > CK > F2. Notably, only the F2 group exhibited a lower total flavor component content after fermentation. The F4 group, which had the highest total flavor component content, showed a 14.4% increase compared with the water fermentation control group. The result also indicated that most *C. fabianii* strains (e.g., F1, F3, F4, F5) could increase the content of chlorophyll degradation products (from 1.65 mg/g to 1.85 ~ 2.06 mg/g) and esters (1.18 mg/g to 1.46 ~ 1.66 mg/g) during fermentation. This result is consistent with previous studies, which have shown that *C. fabianii* can produce ester compounds (van Rijswijck et al., 2017; Liang et al., 2024).

**Figure 3:**
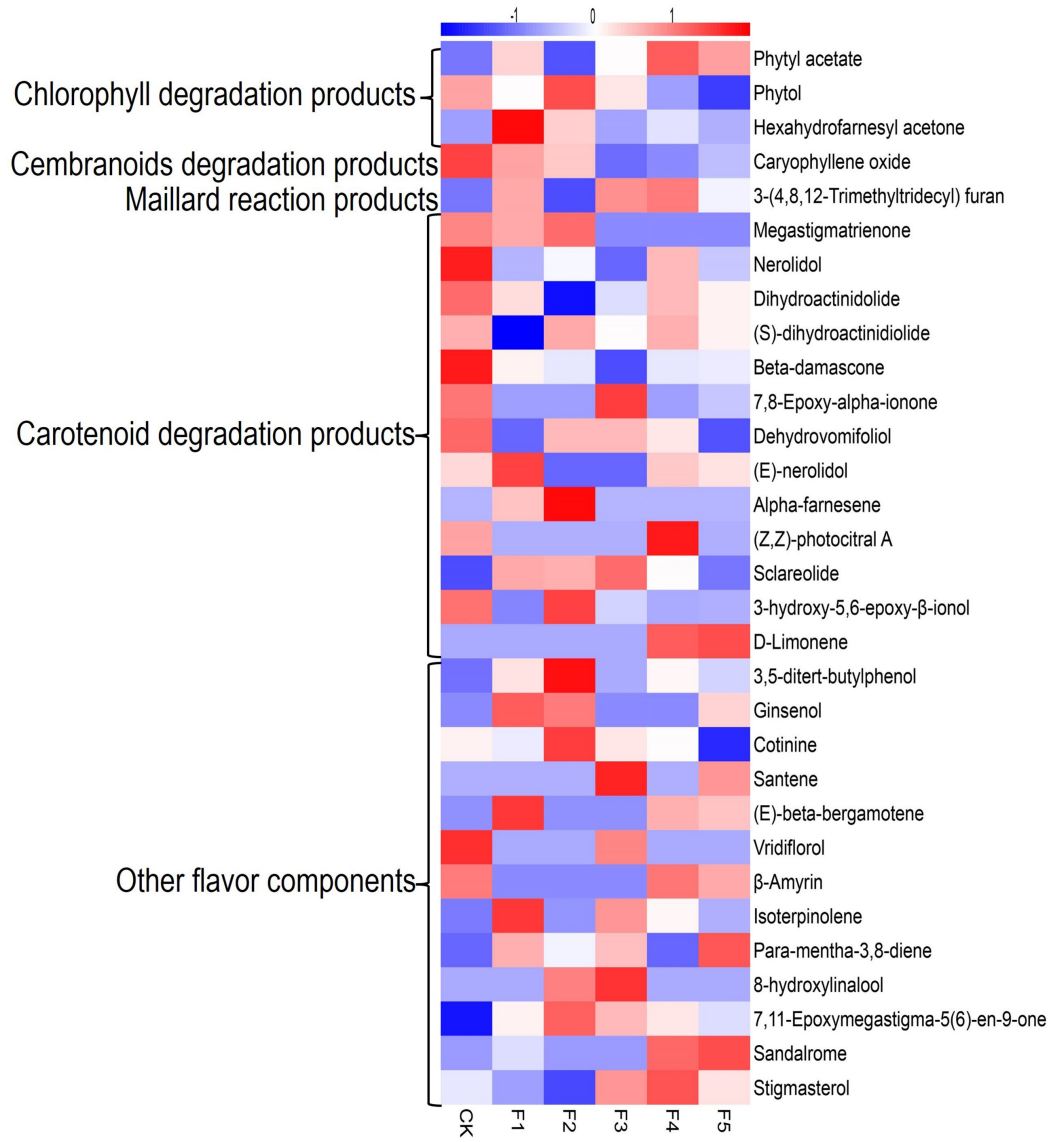
Heatmap of flavor component in different fermentation groups. CK represents water fermentation control group, F1 ~ F5 represent different *C. fabianii* fermentation groups.

Figure 3 illustrates the changes of flavor components detected in different fermentation groups. The CK group exhibits higher levels in several carotenoid degradation products, whereas, the *C. fabianii* groups possess higher content in other flavor components. The F4 group, which exhibited the highest total flavor content, contained 15 flavor components with higher content than the control group, including phytyl acetate, sandalrome, sclareolide, D-Limonene, (E)-beta-bergamotene, 3,5-di-tert-butyl phenol, and 7,11-Epoxymegastigma-5(6)-en-9-one. Among these compounds, phytyl acetate is particularly noteworthy. It serves as both a chlorophyll degradation product and an ester compound. Compared to the control group, the concentration of phytyl acetate was higher in most *C. fabianii* fermentation groups, except for strain F2. Particularly, the F4 fermentation group showed a 41% increase in phytyl acetate over the control group. The above results suggest that the aroma and ester-producing properties of *C. fabianii* contribute to enhancing the flavor component content in CTLs through fermentation. Strain F4, in particular, produces more flavor components and has the potential to improve CTL quality.

### 3.3 Comparative analysis of major chemical compositions

**Figure 4:**
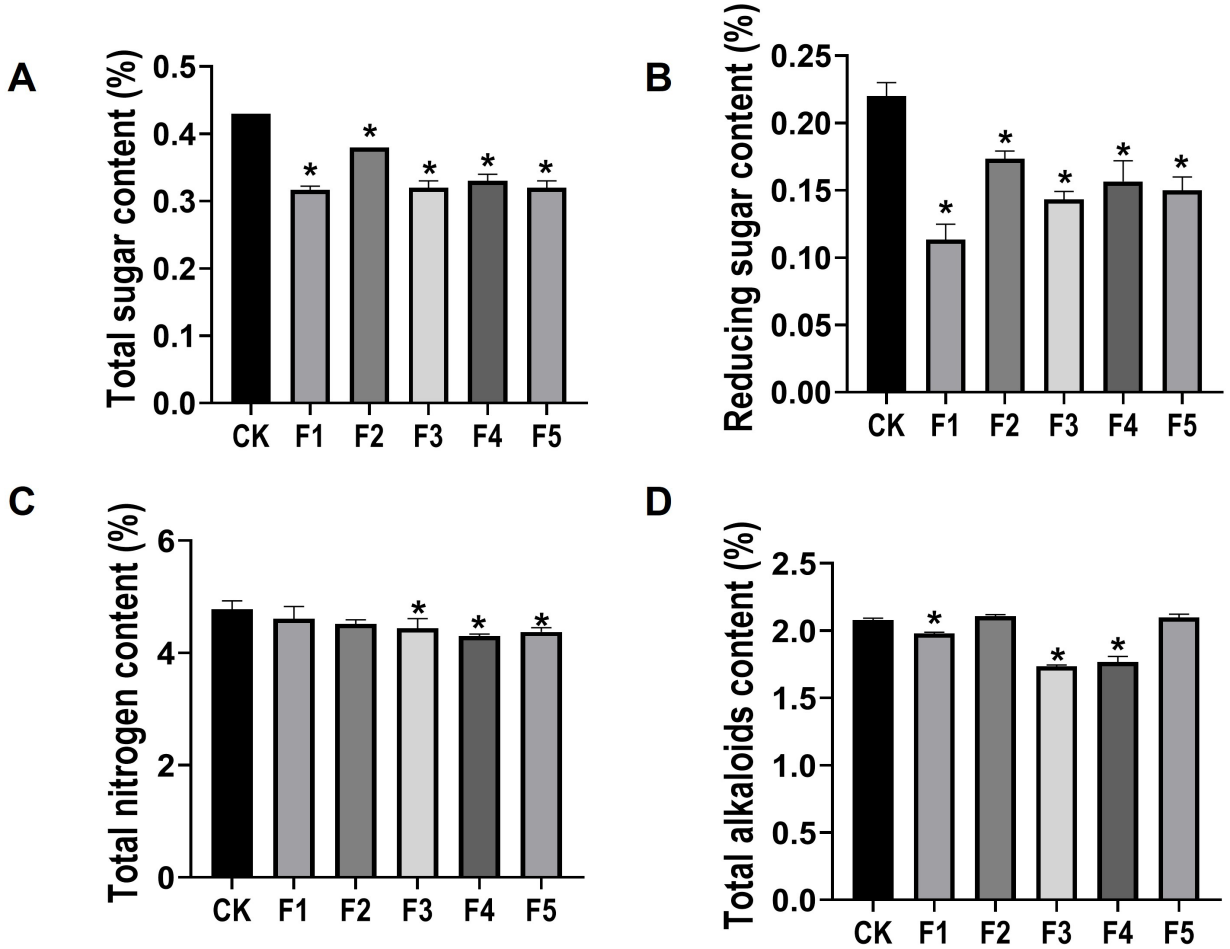
The chemical compositions of CTLs in different fermentation group. **(A)** Total sugar content; **(B)** Reducing sugar content; **(C)** Total nitrogen content; **(D)** Total alkaloids content. CK represents water fermented control group, F1 ~ F5 represent different *C. fabianii* fermentation groups. Asterisks indicate significant differences between CK and *C. fabianii* fermentation groups (Dunnett’s test, *p* < 0.05).

The intrinsic quality of CTLs is influenced by major chemical compositions such as total nitrogen, total sugar, total alkaloids, and reducing sugar. To determine whether *C. fabianii* strains can enhance the intrinsic quality of CTLs during fermentation, we performed continuous flow analysis to monitor changes in these major chemical compositions. Results (Figure 4) showed that strain F3 and F4 significantly reduced four major chemical compositions in CTLs after fermentation, including total alkaloids, total sugar, reducing sugar and total nitrogen. Furthermore, compared with the water fermentation control group, all five isolated *C. fabianii* fermentation groups showed a significant decrease in both total sugar (Figure 4A) and reducing sugar content (Figure 4B). Since total sugar and reducing sugar are crucial precursors of neutral aroma-enhancing compounds (Huang et al., 2024), their reduction in *C. fabianii* fermentation groups suggests an acceleration of biochemical processes. These processes may involve the conversion of sugars, like sucrose and glucose, into flavor compounds through degradation, oxidation, and esterification (Forney and Song, 2017).

High protein content in CTLs can lead to bitterness, irritation, and excessively strong smoke intensity during combustion (Zhang W. et al., 2023), which negatively impacts CTL quality. Since total nitrogen content is a reliable indicator of protein level (Boulos et al., 2020), the total nitrogen content of *C. fabianii* fermented CTLs was detected (Figure 4C). The F3, F4, and F5 groups showed a significant decrease in total nitrogen content compared to the control group, with reductions of 7.11, 10.11, and 8.58%, respectively. This result shows that certain *C. fabianii* strains possess the capacity for utilizing nitrogen-containing substances such as proteins. Furthermore, the amino acids produced by protein degradation can act as precursors of the Maillard reaction, and enhance aromatic compound generation during cigar combustion (Liu et al., 2022).

Excessive alkaloids in cigars can not only cause bitterness and irritation during smoking but also are associated with numerous health risks (Jia et al., 2023; Xue et al., 2023). Figure 4D shows that, strain F1, F3, and F4 significantly reduce the total alkaloids content in CTLs by 4.8, 16.5, and 14.9%, respectively, compared with the control group. The degradation of alkaloids is commonly believed to diminish the harshness of tobacco leaves, resulting in a milder and smoother taste, which contributes to the CTL quality improvement (Xue et al., 2023).

Taken together, post-fermentation, strain F4 exhibited the most significant reduction in total nitrogen (Figure 4C) and a near-maximal decrease in total alkaloids (Figure 4D). Considering the results of flavor components analysis, strain F4 also showed the highest total flavor content (Figure 2A). The above results indicate that strain F4 could be a promising microbial fermentation agent capable of reducing irritation and increasing aroma. Therefore, we chose the strain F4 fermented group for subsequent sensory evaluation.

### 3.4 Sensory evaluation

**Figure 5:**
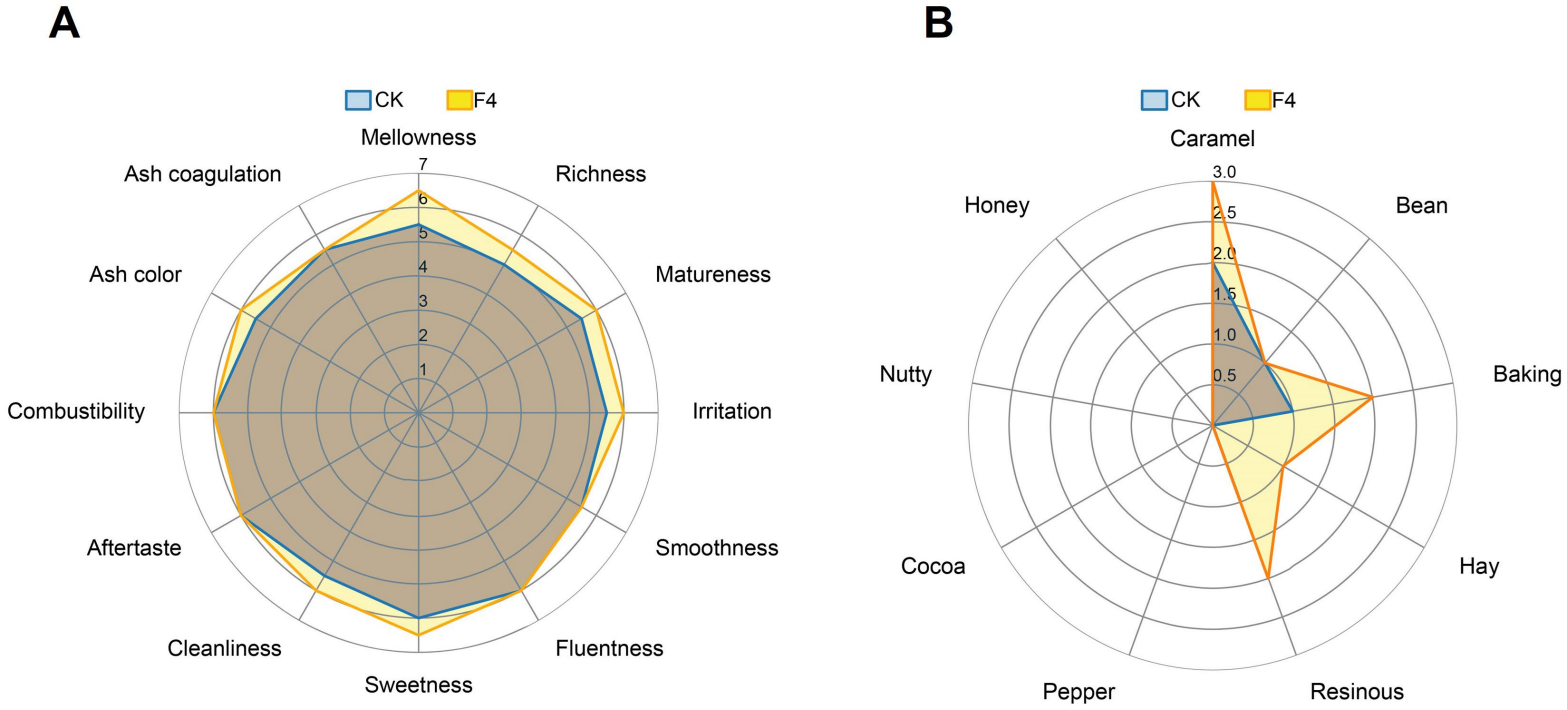
Sensory evaluation radar plots of CTLs fermented by water and *C. fabianii* strain F4. **(A)** Quality characteristics. **(B)** Flavor characteristics. CK represents water fermented group, F4 represents *C. fabianii* fermented group.

Sensory evaluation is the most effective and definitive method to assess CTL fermentation effect and overall cigar quality. To verify that the isolated *C. fabianii* strain F4 can enhance CTL quality through fermentation, we conducted sensory evaluations on CTLs fermented by strain F4, using water-fermented CTLs as a control. The results, illustrated in Figure 5, indicate that the total sensory score of the CTLs fermented with *C. fabianii* strain F4 was higher than that of the control. Specifically, Figure 5A showed that compared with the control group, in terms of irritation and off-flavor, strain F4 could improve cleanliness, matureness and reduce irritation. In terms of aroma quantity, strain F4 could enhance mellowness, richness, and sweetness. As for the aroma profile (Figure 5B), strain F4 could enhance caramel, baking, hay, and resinous flavor of CTLs. The sensory evaluation result was consistent with chemical properties analysis, confirming that *C. fabianii* strain F4 can improve CTL quality through fermentation.

## 4 Discussion

Microorganism fortified fermentation can improve the intrinsic quality of CTLs, reduce harmful compounds, and accelerate the fermentation process (Jia et al., 2023). In this study, to further improve the smoking qualities of CTLs, five *C. fabianii* strains were isolated from CTLs. *C. fabianii* is known for generating ester compounds and being utilized in alcoholic fermentation. The CTLs fermented by five *C. fabianii* strains were analyzed by GC–MS for flavor component detection. As expected, compared with the control group, four *C. fabianii* fermentation groups exhibited higher total flavor content and showed a significant increase in ester content. A previous study showed that CTLs from Sichuan province contain fewer ester compounds compared with other regions such as Hubei and Hainan (Zhu et al., 2024). Since esters provide a sweet and fruity aroma, this strain could potentially be used in Sichuan CTLs fermentation to enhance the ester content and enrich the aroma profile of CTLs produced in Sichuan province. Additionally, we observed a slight decrease in carotenoid degradation products content in the *C. fabianii* fermentation groups. This may be because the inoculation of *C. fabianii* altered the microbial community, thereby reducing carotenoid degradation. Future studies should apply multi-omic approaches to elucidate the underlying mechanisms.

Both flavor component analysis and major chemical composition analysis indicate that *C. fabianii* strain F4 has great potential as a microbial fermentation agent. Compared with the control group, strain F4 increased the total flavor content by 14.4% and increased the chlorophyll degradation products content by 24.8% (Figure 2). The increase in total flavor content enhances aroma intensity and flavor profile. This is consistent with sensory evaluation test (Figure 5A) where the *C. fabianii* strain F4 fermentation group exhibited more mellowness, richness, and sweetness. Meanwhile, the increase of chlorophyll degradation products in F4 fermentation group not only increased the flavor compounds but also reduced the content of irritant chlorophyll, thereby improving CTL quality from both perspectives (Hu et al., 2022). In major chemical composition analysis, since reducing sugars serve as essential precursors in the Maillard reaction, their significant decrease during F4 strain fermentation might contribute to the increasement of Maillard reaction products from 0.011 mg/g to 0.019 mg/g (Hu et al., 2022). Furthermore, *C. fabianii* strain F4 decreased the total alkaloids content and total nitrogen content by 14.9 and 10.11%, respectively, compared to the water fermented group. Excessive alkaloids and proteins in CTLs can cause irritation and bitterness. Reduction of their content via strain F4 fermentation could improve the cleanliness, matureness and reduce irritation of CTLs, which has been confirmed by sensory evaluation. *C. fabianii* strain F4 fermented CTLs showed an increase in caramel, baking, hay, and resinous aromas (Figure 5B). This may be due to its ability to increase flavor compounds such as sandalrome (woody, sandalwood), phytyl acetate (waxy, fruity), D-Limonene (sweet, citrus), 3,5-di-tert-butyl-phenol (caramel and smoky).

All the above results indicate that *C. fabianii* strain F4 can enhance CTL flavor richness and reduce irritation through fermentation. Moreover, as a biotin-prototrophic yeast, *C. fabianii* can rapidly grow in the absence of biotin (Wronska et al., 2020). Co-fermentation of *Saccharomyces cerevisiae* with *C. fabianii* results in beer with a more complex aroma profile (van Rijswijck et al., 2019). Therefore, future research should analyze the microbial community structure and flavor compound variations during *C. fabianii* fermentation. This will help identify and isolate microorganisms capable of co-fermenting with *C. fabianii* to further improve CTL fermentation quality.

## 5 Conclusion

Microorganisms fortified fermentation is one of the most important methods for improving CTL quality. In this study, we isolated five *C. fabianii* strains from CTLs. Our analysis of flavor components and major chemical compositions revealed that most *C. fabianii* strains demonstrate multiple beneficial capabilities during fermentation. These strains can increase total flavor content, decrease total alkaloids content and total nitrogen content during fermentation. Among them, *C. fabianii* strain F4 exhibited the most remarkable fermentation effect. Compared with the control group, strain F4 fermentation group showed a 14.4% increase in total flavor component content. Furthermore, strain F4 also reduced the total alkaloids content by 14.9% and total nitrogen by 10.11% compared with the control group. These reductions could reduce the irritation of CTLs during combustion. The subsequent sensory evaluation also demonstrated that *C. fabianii* strain F4 could improve mellowness, richness, sweetness, and reduce irritation in the fermented CTLs. These results suggest that *C. fabianii* strain F4 has great potential as a microbial fermentation agent for improving the CTLs quality, providing a new approach for the cigar industry to produce higher-quality products.

## Data Availability

The raw data supporting the conclusions of this article will be made available by the authors, without undue reservation.
